# Supplementary material for: Flow coupling between active and passive fluids across water–oil interfaces
Source: Sci Rep. 2021 Jul 7;11:13965. doi: 10.1038/s41598-021-93310-9 (PMC8263611; doi:10.1038/s41598-021-93310-9)
Supplement: Supplementary file 1 — Supplementary Information 1. [file 41598_2021_93310_MOESM1_ESM.pdf]

Supplementary Information:
Flow coupling between active and passive fluids across water–oil interfaces

Yen-Chen Chen[1], Brock Jolicoeur[2], Chih-Che Chueh[3], and Kun-Ta Wu[1,2,4,*]

[1]Department of Mechanical Engineering, Worcester Polytechnic Institute, Worcester, Massachusetts 01609, USA
[2]Department of Physics, Worcester Polytechnic Institute, Worcester, Massachusetts 01609, USA
[3]Department of Aeronautics and Astronautics, National Cheng Kung University, Tainan 701, Taiwan
[4]The Martin Fisher School of Physics, Brandeis University, Waltham, Massachusetts 02454, USA
[*]Corresponding: kwu@wpi.edu

Table of Contents

Supplementary Discussion S1: Influence of the ceiling geometry on intradroplet active fluid behavior 2
Supplementary Fig. S1 2
Supplementary Fig. S2 3
Supplementary Fig. S3 4
Supplementary Discussion S2: Modeling active droplet systems with a continuum simulation 5
Supplementary Table S1 9
Supplementary Discussion S3: Shear stress coupling across water–oil interface 10
Supplementary Fig. S4 10
Supplementary Discussion S4: The role of interfacial properties on flow coupling 12
Supplementary Fig. S5 12
Supplementary Fig. S6 14
Supplementary Discussion S5: Formation of two-dimensional nematic layers at water–oil interfaces 15
Supplementary Fig. S7 15
Supplementary Fig. S8 17
Supplementary Fig. S9 18
Supplementary Video S1 19
Supplementary Video S2 19
Supplementary Video S3 19
Supplementary Video S4 19
Supplementary References 20

Supplementary Discussion S1: Influence of the ceiling geometry on intradroplet active fluid behavior

Compressed active droplets self-propel,[1-4] which could prevent us from observing intradroplet flows for long durations with a fixed observation window. Therefore, we fixed the droplet by curving the ceiling of the fluidic device into a dome-like shape and then drilled a thin well at the dome center to reinforce droplet immobilization (Supplementary Fig. S1a). However, these shape modifications change the droplet shape, which might influence the self-organization of active fluids in droplets. Here, we examined how the intradroplet active fluid flows were influenced by the geometric parameters of the ceiling (i.e., the well dimensions and dome shape).

Influence of well dimensions. First, we investigated the role of the well geometry on intradroplet flows. The well was cylindrical; its geometry depended on its height (or depth) and radius. To examine how these parameters influenced intradroplet flows, we measured the circulation order parameter (COP) within a droplet while varying the well radius (Supplementary Fig. S2a) and depth (Supplementary Fig. S2b) separately. Across our explored parameters, our measured COPs remained steady (fluctuating between 0.4 and 0.6), which suggests that the well geometry did not play a significant role in the formation of intradroplet circulatory flows. In this study, we chose a well geometry (depth 0.2 mm, radius 1 mm) whose

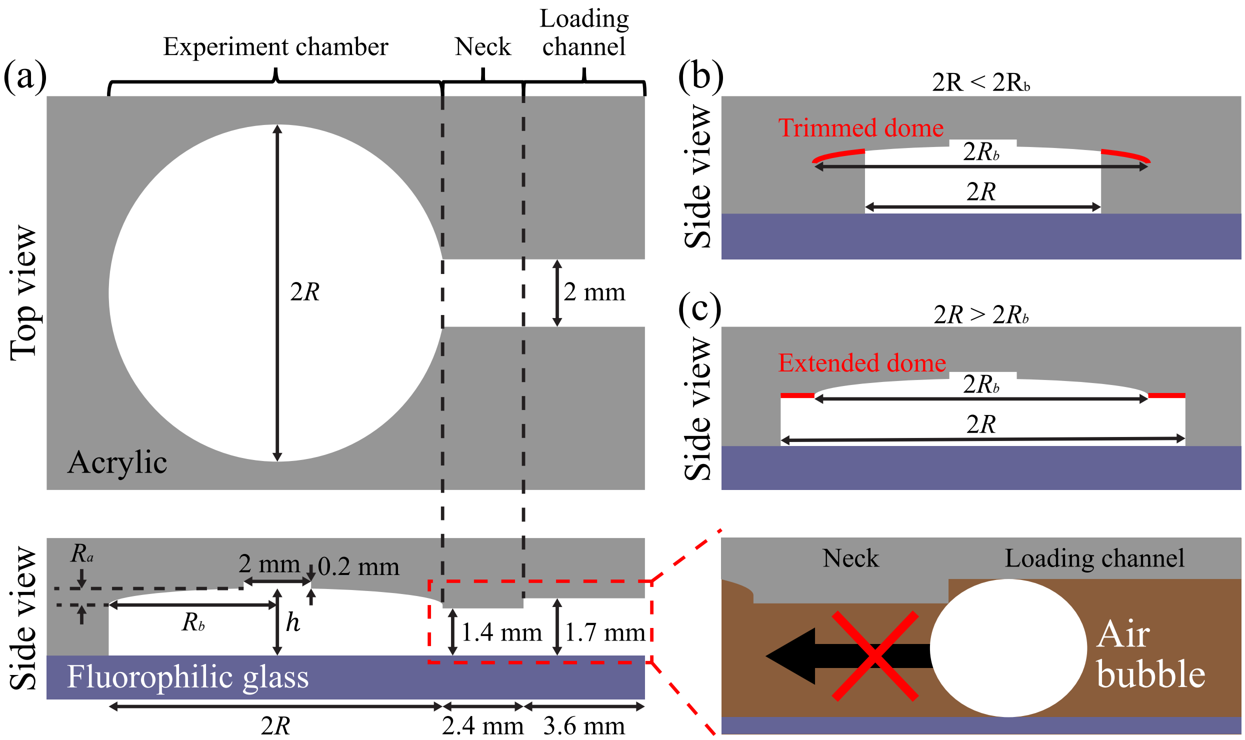

Supplementary Fig. S1: Millifluidic device for compressing an active fluid droplet. (a) The device contained a cylinder-like chamber to contain the oil and droplet, which were loaded via the loading channel. The channel was connected to the chamber via a neck to keep air bubbles out of the chamber (close-up). To fix the droplet in the chamber center, the chamber ceiling was curved into a half-oblate spheroidal shape whose semi-axes were R_a and R_b, and a shallow well was drilled at the ceiling center. Close-up: An air-in-oil bubble in the loading channel could not spontaneously enter the chamber because of the smaller opening of the neck. (b) To fit a half-spheroidal dome into a smaller chamber ($2R < 2R_b$), the dome was trimmed (red curves). (c) Conversely, to match a dome to a larger chamber ($2R > 2R_b$), the dome was extended horizontally (red lines).

COP could not be distinguished from the case without a well (within error bars). We expected that using this well on the ceilings could reinforce droplet immobilization while minimizing the well's influence on intradroplet active fluid flows.

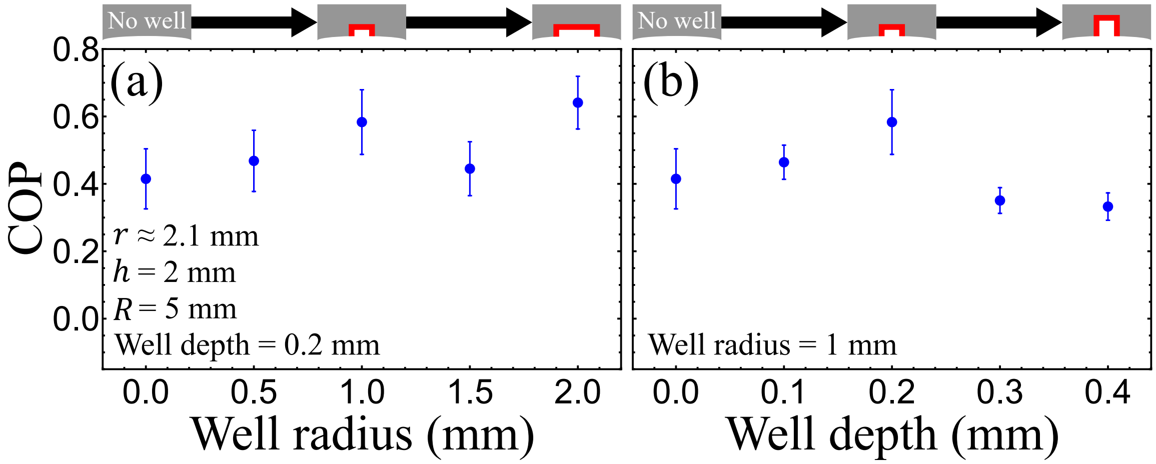

Supplementary Fig. S2: Intradroplet circulatory flows were not significantly influenced by the well dimensions. Geometries of the half-spheroidal domes were fixed (R_a = 0.5 mm, R_b = 5 mm). Varying either the well radius (panel a) or the well depth (panel b) did not significantly alter the circulation order parameter (COP), which suggests that the wells did not play a substantial role in intradroplet flows. Each error bar represents the standard deviation of the time-averaged COP.

Influence of dome shape. Next, we characterized how the intradroplet active fluid flows were influenced by the dome shape. The dome was shaped into a half-oblate spheroid whose geometry depended on its vertical and horizontal semi-axes (Supplementary Fig. S1). To characterize how the dome geometry influenced intradroplet circulatory flows, we systematically varied the vertical semi-axes R_a = 0.25–1.5 mm while maintaining the horizontal semi-axes R_b = R = 5 mm (Supplementary Fig. S1a) and then measured the corresponding COPs of intradroplet flows (Supplementary Fig. S3). Our measurements showed that circulatory flows persisted when the vertical semi-axes were shorter than ~1 mm (COP > 0.4); lengthening the vertical semi-axes longer than this limit weakened the formation of circulatory flows (COP ≤ 0.4). This result suggests that the dome shape influences the formation of intradroplet flows, but this influence was limited to largely curved dome ($R_a \gtrsim$ 1 mm). To minimize the influence of the dome while immobilizing the droplets, we adopted half-spheroidal domes with a vertical semi-axis of R_a = 0.25–0.5 mm and a horizontal semi-axis of R_b = 3.5–5 mm.

In summary, observing the intradroplet active fluid flows over a long duration required compressing the droplets with a curved ceiling. However, the curved ceiling influenced the intradroplet fluid flows. To minimize this influence, we chose the ceiling shape that would not suppress development of circulatory flows but was sufficiently curved to fix the droplets. This arrangement provided a stationary active droplet that allowed us to investigate how the formation of intradroplet circulatory flows was controlled by other geometric parameters such as the droplet radius and oil layer thickness (Figs. 1–3).

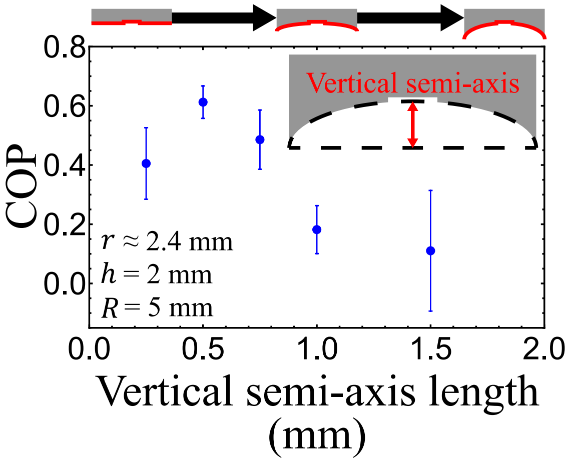

Supplementary Fig. S3: Intradroplet circulatory flows were influenced by the dome shape. The well geometry was fixed (radius 1 mm, depth 0.5 mm), and the domes were half-oblate spheroids with a horizontal semi-axis of 5 mm and vertical semi-axes ranging from 0.25 to 1.5 mm. Lengthening the vertical semi-axes increased the dome curvature. Such increases in curvature sharpened the droplet shape and suppressed development of circulatory flows when vertical semi-axes were longer than ~1 mm (circulatory order parameter [COP] ≤ 0.4); for the semi-axes shorter than this limit, circulatory flows were stable, which suggests that domes with a larger curvature had a greater influence on intradroplet flows. Thus, we selected a slightly curved dome ($R_a = 0.5$ mm, $R_b = 5$ mm) to compress the droplets. Each error bar represents the standard deviation of the time-averaged COP.

Supplementary Discussion S2: Modeling active droplet systems with a continuum simulation

To test whether an existing active fluid model was capable of describing the influences of active fluid–oil coupling on intradroplet active fluid flows,[3,5-25] we adopted the active droplet model established by Gao *et al.* because this model closely matched our experimental system which involved a microtubule-based active fluid in a water-in-oil droplet.[3] The model applied a cylindrical no-slip boundary (radius R, height h) with a curved ceiling, which was identical to the confinement geometry in the experiments (Fig. 1b). The boundary was filled with oil (density ρ_o, dynamic viscosity μ_o) surrounding a concentric cylindrical active fluid (radius r, density ρ_a, dynamic viscosity μ_a) modeled as a compressed water-in-oil droplet. The water–oil interface was modeled as variations of a phase function:

$$c = H\left(1 - \frac{\sqrt{x^2 + y^2}}{r}\right), \qquad \text{(Eq. S1)}$$

where H is the Heaviside step function with $c\left(\sqrt{x^2 + y^2} \le r\right) = 1$ representing the active fluid and $c\left(\sqrt{x^2 + y^2} > r\right) = 0$ representing the oil. To speed up the simulations and to match our experimental arrangement for immobilizing the droplets, we fixed the water–oil interface so the phase function was kept constant, rather than evolving as in the Cahn–Hilliard model.[26] The phase function varied at the water–oil interface, which induced interfacial tension forces $\boldsymbol{T} \equiv (\gamma K/\epsilon)\boldsymbol{\nabla} c$, where γ is the interfacial tension, ϵ is the interface thickness, and $K \equiv c(c-1)(c-1/2) - \epsilon^2\nabla^2 c$ is the chemical potential that characterizes the phase variation within the interfacial region.[27] Within the interface, active fluid generated active stress ($\boldsymbol{\sigma}_a$) to induce self-driven flows ($\boldsymbol{u}$) in incompressible fluids ($\boldsymbol{\nabla} \cdot \boldsymbol{u} = 0$) that were governed by the Navier–Stokes equation:

$$\rho\left(\frac{\partial \boldsymbol{u}}{\partial t} + \boldsymbol{u} \cdot \boldsymbol{\nabla} \boldsymbol{u}\right) = \nabla \cdot [-p\boldsymbol{I} + \mu(\boldsymbol{\nabla} \boldsymbol{u} + \boldsymbol{\nabla} \boldsymbol{u}^T)] + \boldsymbol{F}, \qquad \text{(Eq. S2)}$$

where ρ is the fluid density, p is the fluid pressure, μ is the dynamic viscosity of the fluid, and $\boldsymbol{F} \equiv \boldsymbol{T} + \nabla \cdot \boldsymbol{\sigma}_a$ is the net body force. Calculating the net body force required determining the active stress, which was exerted by the extensile microtubule bundles in the experiment. Here, we modeled each extensile bundle as a self-elongating rod whose center of mass is $\boldsymbol{x} = (x, y, z)$ orienting at $\boldsymbol{p} = (\sin\theta\cos\varphi, \sin\theta\sin\varphi, \cos\theta)$. The configurations of these rods were described with a mean-field probability distribution function, $\Psi(\boldsymbol{x}, \boldsymbol{p}, t)$, normalized as $\int_{\boldsymbol{p}} \Psi d\boldsymbol{p} = 1$. To conserve probability, the distribution function satisfied the Smoluchowski equation as:

$$\frac{\partial \Psi}{\partial t} + \boldsymbol{\nabla} \cdot (\dot{\boldsymbol{x}}\Psi) + \boldsymbol{\nabla}_{\boldsymbol{p}} \cdot (\dot{\boldsymbol{p}}\Psi) = 0, \qquad \text{(Eq. S3)}$$

where $\boldsymbol{\nabla}_{\boldsymbol{p}} \equiv \partial(\boldsymbol{I} - \boldsymbol{pp})/\partial\boldsymbol{p}$ is the surface derivative on the unit sphere.[10,12,28,29] Considering that these rods were only advected by fluid flows, the translational flux of the rods was determined as $\dot{\boldsymbol{x}} = \boldsymbol{u} - D_T\boldsymbol{\nabla}\ln\Psi$, where D_T is the translational diffusion coefficient. The rotational flux induced by the fluid velocity gradient is $\dot{\boldsymbol{p}} = (\boldsymbol{I} - \boldsymbol{pp}) \cdot (\boldsymbol{\nabla}\boldsymbol{u} + 2\zeta\boldsymbol{D}) \cdot \boldsymbol{p} - D_R\boldsymbol{\nabla}_{\boldsymbol{p}}\ln\Psi$, where ζ is the mean-field torque strength and D_R is the rotational diffusion coefficient.[12,30-32] These equations describe the coupling between the fluid flows and the rods' translational and rotational distributions, but solving these equations was computationally expensive. To reduce the computation load, the Smoluchowski equation was coarse-grained as

$$\frac{\partial \boldsymbol{D}}{\partial t} - D_T\Delta\boldsymbol{D} + \boldsymbol{u} \cdot \boldsymbol{\nabla}\boldsymbol{D} = 4\zeta(\boldsymbol{D} \cdot \boldsymbol{D} - \boldsymbol{D}\colon\boldsymbol{S}) + \boldsymbol{\nabla}\boldsymbol{u} \cdot \boldsymbol{D} + \boldsymbol{D} \cdot \boldsymbol{\nabla}\boldsymbol{u}^T - 2\boldsymbol{E}\colon\boldsymbol{S} - 6D_R\left(\boldsymbol{D} - \frac{\boldsymbol{I}}{3}\right), \qquad \text{(Eq. S4)}$$

where $\boldsymbol{E} \equiv \frac{\nabla \boldsymbol{u}+\nabla \boldsymbol{u}^T}{2}$ is the strain rate tensor, $\boldsymbol{D} \equiv \int_{\boldsymbol{p}} \boldsymbol{pp}\Psi \boldsymbol{dp}$ is the orientational average of the second moment tensor of the rod orientation $\boldsymbol{pp}$, and $\boldsymbol{S} \equiv \boldsymbol{DD}$.[29,33] The coarse-grained Smoluchowski equation allowed for determining the configuration of the self-elongating rods, which generated an active stress that was modeled to be proportional to the local rod orientational order:[21] $\boldsymbol{\sigma}_a = \alpha c \boldsymbol{D}$, where α is the activity coefficient ($\alpha > 0$ represents contracting rods and $\alpha < 0$ represents elongating rods) and $\boldsymbol{D} \equiv \int_{\boldsymbol{p}} \boldsymbol{pp}\Psi \boldsymbol{dp}$ represents the orientational order of the rods.[34] The active stress drove the fluids whose flows were determined via the Navier–Stokes equation (Eq. S2), which in return rearranged the rod configurations and reformulated the active stress via the coarse-grained Smoluchowski equation (Eq. S4). Both equations formed a feedback loop to simultaneously evolve the fluid flows, rod configurations, and active stresses.

To solve these equations and to develop a simulation platform for modeling our active droplet systems, we expressed both equations in explicit forms that were fed into COMSOL Multiphysics™, which solved the equations by the finite element method.[35-39] To feed in the Navier–Stokes equation (Eq. S2), we utilized the template of the 3D laminar flow model in the software. This template considers incompressible fluids ($\nabla \cdot \boldsymbol{u} = 0$) governed by the Navier–Stokes equation with a net body force that can be expressed as

$$\boldsymbol{F} = \begin{bmatrix} F_x \\ F_y \\ F_z \end{bmatrix} = \alpha \begin{bmatrix} c\left(\frac{\partial D_{xx}}{\partial x} + \frac{\partial D_{yx}}{\partial y} + \frac{\partial D_{zx}}{\partial z}\right) + D_{xx}\frac{\partial c}{\partial x} + D_{yx}\frac{\partial c}{\partial y} + D_{zx}\frac{\partial c}{\partial z} \\ c\left(\frac{\partial D_{xy}}{\partial x} + \frac{\partial D_{yy}}{\partial y} + \frac{\partial D_{zy}}{\partial z}\right) + D_{xy}\frac{\partial c}{\partial x} + D_{yy}\frac{\partial c}{\partial y} + D_{zy}\frac{\partial c}{\partial z} \\ c\left(\frac{\partial D_{xz}}{\partial x} + \frac{\partial D_{yz}}{\partial y} + \frac{\partial D_{zz}}{\partial z}\right) + D_{xz}\frac{\partial c}{\partial x} + D_{yz}\frac{\partial c}{\partial y} + D_{zz}\frac{\partial c}{\partial z} \end{bmatrix} + \frac{\gamma K}{\epsilon} \begin{bmatrix} \frac{\partial c}{\partial x} \\ \frac{\partial c}{\partial y} \\ \frac{\partial c}{\partial z} \end{bmatrix}, \quad \text{(Eq. S5)}$$

where $\frac{\partial c}{\partial x_i} = \frac{-x_i \delta\left(r-\sqrt{x^2+y^2}\right)}{\sqrt{x^2+y^2}}$, $K = c(c-1)\left(c-\frac{1}{2}\right) - \epsilon^2\left[\delta'\left(r-\sqrt{x^2+y^2}\right) - \frac{\delta\left(r-\sqrt{x^2+y^2}\right)}{\sqrt{x^2+y^2}}\right]$, and δ is the Dirac delta function. To include the coarse-grained Smoluchowski equation (Eq. S4), we rearranged the equation as:

$$\frac{\partial \boldsymbol{D}}{\partial t} - D_T \Delta \boldsymbol{D} + \begin{bmatrix} u_x \\ u_y \\ u_z \end{bmatrix} \cdot \nabla \boldsymbol{D} = -\begin{bmatrix} a_{xx} & a_{xy} & a_{xz} \\ a_{yx} & a_{yy} & a_{yz} \\ a_{zx} & a_{zy} & a_{zz} \end{bmatrix} \circ \boldsymbol{D} + \begin{bmatrix} f_{xx} & f_{xy} & f_{xz} \\ f_{yx} & f_{yy} & f_{yz} \\ f_{zx} & f_{zy} & f_{zz} \end{bmatrix}, \quad \text{(Eq. S6)}$$

where

$$\begin{aligned} a_{xx} = {} & 4\zeta\left(D_{xx}^2 + D_{yy}^2 + D_{zz}^2 + 2D_{xy}D_{yx} + 2D_{xz}D_{zx} + 2D_{yz}D_{zy} - D_{xx}\right) \\ & + 2\left[(D_{xx}-1)\frac{\partial u_x}{\partial x} + D_{yy}\frac{\partial u_y}{\partial y} + D_{zz}\frac{\partial u_z}{\partial z}\right] + \left(D_{xy} + D_{yx}\right)\left(\frac{\partial u_x}{\partial y} + \frac{\partial u_y}{\partial x}\right) \\ & + (D_{xz} + D_{zx})\left(\frac{\partial u_x}{\partial z} + \frac{\partial u_z}{\partial x}\right) + \left(D_{yz} + D_{zy}\right)\left(\frac{\partial u_y}{\partial z} + \frac{\partial u_z}{\partial y}\right) + 6D_R, \end{aligned}$$

$$\begin{aligned} a_{yy} = {} & 4\zeta\left(D_{xx}^2 + D_{yy}^2 + D_{zz}^2 + 2D_{xy}D_{yx} + 2D_{xz}D_{zx} + 2D_{yz}D_{zy} - D_{yy}\right) \\ & + 2\left[D_{xx}\frac{\partial u_x}{\partial x} + \left(D_{yy}-1\right)\frac{\partial u_y}{\partial y} + D_{zz}\frac{\partial u_z}{\partial z}\right] + \left(D_{xy} + D_{yx}\right)\left(\frac{\partial u_x}{\partial y} + \frac{\partial u_y}{\partial x}\right) \\ & + (D_{xz} + D_{zx})\left(\frac{\partial u_x}{\partial z} + \frac{\partial u_z}{\partial x}\right) + \left(D_{yz} + D_{zy}\right)\left(\frac{\partial u_y}{\partial z} + \frac{\partial u_z}{\partial y}\right) + 6D_R, \end{aligned}$$

$$a_{zz} = 4\zeta\left(D_{xx}^2 + D_{yy}^2 + D_{zz}^2 + 2D_{xy}D_{yx} + 2D_{xz}D_{zx} + 2D_{yz}D_{zy} - D_{zz}\right)$$
$$+ 2\left[D_{xx}\frac{\partial u_x}{\partial x} + D_{yy}\frac{\partial u_y}{\partial y} + (D_{zz} - 1)\frac{\partial u_z}{\partial z}\right] + \left(D_{xy} + D_{yx}\right)\left(\frac{\partial u_x}{\partial y} + \frac{\partial u_y}{\partial x}\right)$$
$$+ (D_{xz} + D_{zx})\left(\frac{\partial u_x}{\partial z} + \frac{\partial u_z}{\partial x}\right) + \left(D_{yz} + D_{zy}\right)\left(\frac{\partial u_y}{\partial z} + \frac{\partial u_z}{\partial y}\right) + 6D_R,$$

$$a_{xy} = a_{yx} = 4\zeta\left(D_{xx}^2 + D_{yy}^2 + D_{zz}^2 + 2D_{xy}D_{yx} + 2D_{xz}D_{zx} + 2D_{yz}D_{zy} - D_{xx} - D_{yy}\right)$$
$$+ 2\left[\left(D_{xx} - \frac{1}{2}\right)\frac{\partial u_x}{\partial x} + \left(D_{yy} - \frac{1}{2}\right)\frac{\partial u_y}{\partial y} + D_{zz}\frac{\partial u_z}{\partial z}\right] + \left(D_{xy} + D_{yx}\right)\left(\frac{\partial u_x}{\partial y} + \frac{\partial u_y}{\partial x}\right)$$
$$+ (D_{xz} + D_{zx})\left(\frac{\partial u_x}{\partial z} + \frac{\partial u_z}{\partial x}\right) + \left(D_{yz} + D_{zy}\right)\left(\frac{\partial u_y}{\partial z} + \frac{\partial u_z}{\partial y}\right) + 6D_R,$$

$$a_{xz} = a_{zx} = 4\zeta\left(D_{xx}^2 + D_{yy}^2 + D_{zz}^2 + 2D_{xy}D_{yx} + 2D_{xz}D_{zx} + 2D_{yz}D_{zy} - D_{xx} - D_{zz}\right)$$
$$+ 2\left[\left(D_{xx} - \frac{1}{2}\right)\frac{\partial u_x}{\partial x} + D_{yy}\frac{\partial u_y}{\partial y} + \left(D_{zz} - \frac{1}{2}\right)\frac{\partial u_z}{\partial z}\right] + \left(D_{xy} + D_{yx}\right)\left(\frac{\partial u_x}{\partial y} + \frac{\partial u_y}{\partial x}\right)$$
$$+ (D_{xz} + D_{zx})\left(\frac{\partial u_x}{\partial z} + \frac{\partial u_z}{\partial x}\right) + \left(D_{yz} + D_{zy}\right)\left(\frac{\partial u_y}{\partial z} + \frac{\partial u_z}{\partial y}\right) + 6D_R,$$

$$a_{yz} = a_{zy} = 4\zeta\left(D_{xx}^2 + D_{yy}^2 + D_{zz}^2 + 2D_{xy}D_{yx} + 2D_{xz}D_{zx} + 2D_{yz}D_{zy} - D_{yy} - D_{zz}\right)$$
$$+ 2\left[D_{xx}\frac{\partial u_x}{\partial x} + \left(D_{yy} - \frac{1}{2}\right)\frac{\partial u_y}{\partial y} + \left(D_{zz} - \frac{1}{2}\right)\frac{\partial u_z}{\partial z}\right] + \left(D_{xy} + D_{yx}\right)\left(\frac{\partial u_x}{\partial y} + \frac{\partial u_y}{\partial x}\right)$$
$$+ (D_{xz} + D_{zx})\left(\frac{\partial u_x}{\partial z} + \frac{\partial u_z}{\partial x}\right) + \left(D_{yz} + D_{zy}\right)\left(\frac{\partial u_y}{\partial z} + \frac{\partial u_z}{\partial y}\right) + 6D_R,$$

$$f_{xx} = 4\zeta\left(D_{xy}D_{yx} + D_{xz}D_{zx}\right) + \left(D_{xy} + D_{yx}\right)\frac{\partial u_y}{\partial x} + (D_{xz} + D_{zx})\frac{\partial u_z}{\partial x} + 2D_R,$$

$$f_{yy} = 4\zeta\left(D_{xy}D_{yx} + D_{yz}D_{zy}\right) + \left(D_{xy} + D_{yx}\right)\frac{\partial u_x}{\partial y} + \left(D_{yz} + D_{zy}\right)\frac{\partial u_z}{\partial y} + 2D_R,$$

$$f_{zz} = 4\zeta\left(D_{xz}D_{zx} + D_{yz}D_{zy}\right) + (D_{xz} + D_{zx})\frac{\partial u_x}{\partial z} + \left(D_{yz} + D_{zy}\right)\frac{\partial u_y}{\partial z} + 2D_R,$$

$$f_{xy} = 4\zeta D_{xz}D_{zy} + D_{xx}\frac{\partial u_x}{\partial y} + D_{xz}\frac{\partial u_z}{\partial y} + D_{yy}\frac{\partial u_y}{\partial x} + D_{zy}\frac{\partial u_z}{\partial x},$$

$$f_{yx} = 4\zeta D_{yz}D_{zx} + D_{xx}\frac{\partial u_x}{\partial y} + D_{zx}\frac{\partial u_z}{\partial y} + D_{yy}\frac{\partial u_y}{\partial x} + D_{yz}\frac{\partial u_z}{\partial x},$$

$$f_{xz} = 4\zeta D_{xy}D_{yz} + D_{xx}\frac{\partial u_x}{\partial z} + D_{xy}\frac{\partial u_y}{\partial z} + D_{yz}\frac{\partial u_y}{\partial x} + D_{zz}\frac{\partial u_z}{\partial x},$$

$$f_{zx} = 4\zeta D_{yx}D_{zy} + D_{xx}\frac{\partial u_x}{\partial z} + D_{yx}\frac{\partial u_y}{\partial z} + D_{zy}\frac{\partial u_y}{\partial x} + D_{zz}\frac{\partial u_z}{\partial x},$$

$$f_{yz} = 4\zeta D_{xz}D_{yx} + D_{yx}\frac{\partial u_x}{\partial z} + D_{yy}\frac{\partial u_y}{\partial z} + D_{xz}\frac{\partial u_x}{\partial y} + D_{zz}\frac{\partial u_z}{\partial y}, \text{ and}$$

$$f_{zy} = 4\zeta D_{xy}D_{zx} + D_{xy}\frac{\partial u_x}{\partial z} + D_{yy}\frac{\partial u_y}{\partial z} + D_{zx}\frac{\partial u_x}{\partial y} + D_{zz}\frac{\partial u_z}{\partial y}.$$

The rearranged equation was fed into the software by means of the built-in stabilized convective diffusion equation. Solving these equations numerically required defining the system domains and the associated boundary conditions. As such, we imported 3D computer-aided designs (SOLIDWORKS) identical to the geometries of our experimental containers (Fig. 1b) as system domains and then imposed to the domain surfaces a no-slip boundary condition: $\boldsymbol{u} = \boldsymbol{0}$. Because the rods were limited within the domain, we also imposed a no-flux boundary condition: $\boldsymbol{n} \cdot \nabla\Psi = 0$, or equivalently $\boldsymbol{n} \cdot \boldsymbol{\nabla D} = \boldsymbol{0}$, where $\boldsymbol{n}$ represents the unit vectors normal to domain surfaces, without enforcing rod orientations at boundaries.[29,33] To evolve the fluid flows and rod configurations, we initialized the fluids as quiescent fluids ($\boldsymbol{u} = \boldsymbol{0}$) under uniform pressure ($p = 0$) with uniformly suspended isotropic rods whose translational and orientational distributions were perturbed with 15 random modes:[10,11]

$$\Psi(\boldsymbol{x}, \boldsymbol{p}, 0) = \frac{1}{4\pi}\left[1 + \sum_{i=1}^{15} \epsilon_i P_i(\boldsymbol{p}) \cos(\boldsymbol{k}_i \cdot \boldsymbol{x} + \varsigma_i)\right], \qquad \text{(Eq. S7)}$$

which determined the initial second moment tensor as

$$\boldsymbol{D}(\boldsymbol{x}, 0) = \int_{\boldsymbol{p}} \boldsymbol{pp}\Psi(\boldsymbol{x}, \boldsymbol{p}, 0) d\boldsymbol{p} = \frac{\boldsymbol{I}}{3} + \frac{1}{4\pi}\sum_{i=1}^{15} \epsilon_i \cos(\boldsymbol{k}_i \cdot \boldsymbol{x} + \varsigma_i) \int_{\boldsymbol{p}} \boldsymbol{pp} P_i(\boldsymbol{p}) d\boldsymbol{p}, \qquad \text{(Eq. S8)}$$

where ϵ_i is the random numbers in $[-0.01, 0.01]$, $\boldsymbol{k}_i$ is the random wave numbers whose components are random numbers in $[\pi, 15\pi]$ mm^{-1}, ς_i is the random phases in $[0, 2\pi]$, and $P_i(\boldsymbol{p}) = \sum_{j=1}^{3}\sum_{k=1}^{4} \xi_{ijk} g_k^j$ is the random polynomials of sine and cosine with ξ_{ijk} as the random numbers in $[0, 1]$ and g_k defined as: $g_1 \equiv \sin(\theta)$, $g_2 \equiv \cos(\theta)$, $g_3 \equiv \sin(\varphi)$, and $g_4 \equiv \cos(\varphi)$. The initialized second moment tensor and fluid flow were evolved for 3 hours with the selected model parameters (Supplementary Table S1). The resulting flow field ($\boldsymbol{u}$) was analyzed to determine the circulation order parameters and flow profiles (Fig. 6), which were compared with experimental outcomes (Figs. 3&5).

Symbol	Description	Value
α	Activity coefficient ($\alpha < 0$: elogating rods; $\alpha > 0$: contracting rods)	–50 Pa
γ	Water–oil interfacial tension[40] at 25 °C	0.072 N/m
ϵ	Thickness of the water–oil interface	50 μm
D_T	Translational diffusion coefficient of rods	0.011 m^2/s
D_R	Rotational diffusion coefficient of rods	10 s^{-1}
ζ	Mean-field torque strength on rods induced by flow velocity gradient	100 s^{-1}
ρ_a	Density of active fluid (96% water) at 25 °C	997 kg/m^3
ρ_o	Density[41] of oil (hydrofluoroether, 3M Novec 7500) at 25 °C	1614 kg/m^3
μ_a	Dynamic viscosity of active fluid (96% water) at 25 °C	0.00089 Pa·s
μ_o	Dynamic viscosity[41] of oil (hydrofluoroether) at 25 °C	0.00124 Pa·s

Supplementary Table S1: Parameters used in the simulations. The activity coefficient, translational diffusion coefficient, and rotational diffusion coefficient were selected to match the flow speeds in the simulation with those in the experiments (~10 μm/s). The mean-field torque strength was selected to be four times larger than the rotational diffusion coefficient ($\zeta > 4D_R$) to enforce strong flow alignment of the rods. The water–oil interfacial tension was approximated as the surface tension of water[40] at 25 °C.

Supplementary Discussion S3: Shear stress coupling across water–oil interface

Flow coupling between active fluid and oil is the consequence of dynamic interactions between the fluids across their interfaces. To advance our understanding of these interactions, we used the simulation to investigate shear stress coupling across interfaces between water (inactive fluid) and oil. We used our established active fluid simulation (Fig. 6), turned off activity of active fluid ($\alpha = 0$), imposed shear stress within the droplet ($r = 2.4$ mm, $h = 2$ mm) by arranging a concentric cylinder (radius 1.2 mm) that rotated at a constant angular velocity ($\omega = 0.015$ s^{-1}) with a surface azimuthal velocity of 18 μm/s, and then evolved the fluid flows until they reached the steady state (Supplementary Fig. S4a). To reveal the role of oil layer thickness in shear stress coupling, we repeated the simulation for various oil layer thickness (Δ = 0.23–9.6 mm) and analyzed the profiles of azimuthal velocity (Supplementary Fig. S4b) and corresponding

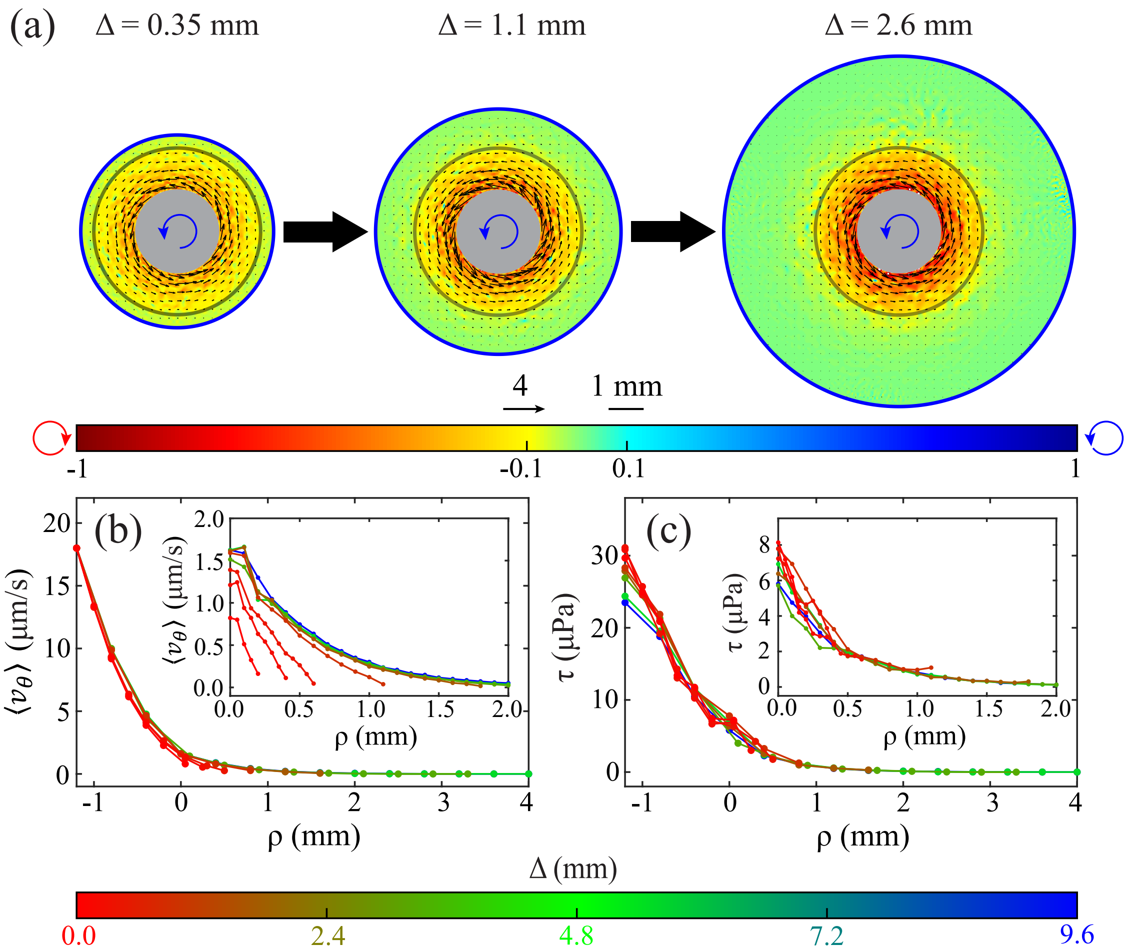

Supplementary Fig. S4: Simulation results show that shear stresses on both sides of water–oil interfaces were coupled with a millimeter–scale coupling length. (a) Velocity fields and vorticity maps (plotted as in Fig. 1c) of three identical water-in-oil droplets (r =2.4 mm, h = 2 mm) surrounded by oil layers of different thicknesses. Within each droplet was a concentric rotating cylinder (angular velocity 0.015 s^{-1}) that generated intradroplet shear stress. (b) Profile of azimuthal velocities for droplets immersed in oil layer thicknesses from 0.23 to 9.6 mm. The horizontal axis represents the radial axis in cylindrical coordinate with the origin shifted to the droplet interface (Fig. 5c inset). Inset: Close-up near the water–oil interface ($\rho = 0$). (c) Profile of corresponding shear stress magnitude $\tau \equiv \left|\langle \mu\rho \frac{d}{d\rho}\left(\frac{v_\theta}{\rho}\right)\rangle\right|$. Inset: Close-up near the water–oil interface.

magnitude of shear stress $\tau \equiv \left|\langle \mu\rho \frac{d}{d\rho}\left(\frac{v_\theta}{\rho}\right)\rangle\right|$, where μ is viscosity of either water or oil depending on the radial coordinate, ρ, and $\langle \quad \rangle$ indicates averaging over θ (Supplementary Fig. S4c). Our analysis showed that the profiles of azimuthal flow depended on oil layer thickness because the imposed no-slip boundary condition at the outer boundary enforced the flow to decay to quiescence at the outer boundary; as such, a thinner oil layer drove the flow to decay more quickly (Supplementary Fig. S4b). Conversely, the profiles of shear stress were nearly independent of oil layer thickness because the stress is permitted to be nonzero at the outer boundary (Supplementary Fig. S4c). The thickness-independent profiles showed that the shear stresses induced within the droplets penetrated the water–oil interface and decayed in oil with a universal millimeter–scale decay length ($L_s \approx 1$ mm). This universal decay length scale of shear stress is consistent with flow coupling length scale observed in our experiments and model (Figs. 3–7), which implies that the flow coupling between active fluid and oil is related to the stress coupling between these two fluids. In active fluid droplets, the shear stress was induced by extensile microtubule bundles; our simulation suggests that this active shear stress could penetrate water–oil interface into the oil to a millimeter–scale depth. When the oil was deeper than this depth ($\Delta > L_s$), the dynamics of extensile bundles were only coupled to a portion of oil near interface; conversely, when the oil was thinner than this depth ($\Delta \lesssim L_s$), the bundle dynamics were coupled to the entire oil layer. Thus, in our experiments and model, we observed that the intradroplet circulatory flows could develop when the thickness of the oil layer was sufficiently large ($\Delta > L_s$) but could be suppressed by the active fluid–oil coupling when the thickness of the oil layer became smaller ($\Delta \lesssim L_s$) (Figs. 3&6).

Supplementary Discussion S4: The role of interfacial properties on flow coupling

We have investigated how the flow coupling between active fluid and oil across the water–oil interfaces influences the self-organization of intradroplet active fluid. As this coupling is across interfaces, it is expected to be influenced by interfacial properties. We used our established simulation platform to investigate how the coupling and intradroplet active fluid flows were affected by two interfacial properties: viscosity contrast and interfacial tension.

Viscosity contrast. To reveal the role of viscosity contrast in the fluid dynamics of the active droplet system (Fig. 6a), we varied the oil viscosity from 0.00089 to 0.124 Pa·s and the viscosity constrast, $\bar{\mu} \equiv \eta_o/\eta_w$, from 1 to 140 and analyzed the time-averaged circulation order parameter (COP; Supplementary Fig. S5a). Our analysis showed that in the simulation of the droplet ($r = 2.4$ mm, $h = 2$ mm) with the thicker oil layer ($\Delta = 2.6$ mm) that supported circulatory flows, viscosity contrast had no significant effect on COP except for the lowest viscosity contrast ($\bar{\mu} = 1$) where active fluid and oil had an identical viscosity (blue dots), whereas the noncirculating droplet immersed in the thinner oil layer ($\Delta = 1.1$ mm) developed circulation as the oil became more viscous (red dots). This result showed that the interfacial viscosity contrast promoted the formation of intradroplet circulatory flows.

To gain deeper insight into this viscosity contrast-aided circulation, we analyzed the cross-correlation length between the active fluid and oil (Supplementary Fig. S5c) and found that the correlation lengths in

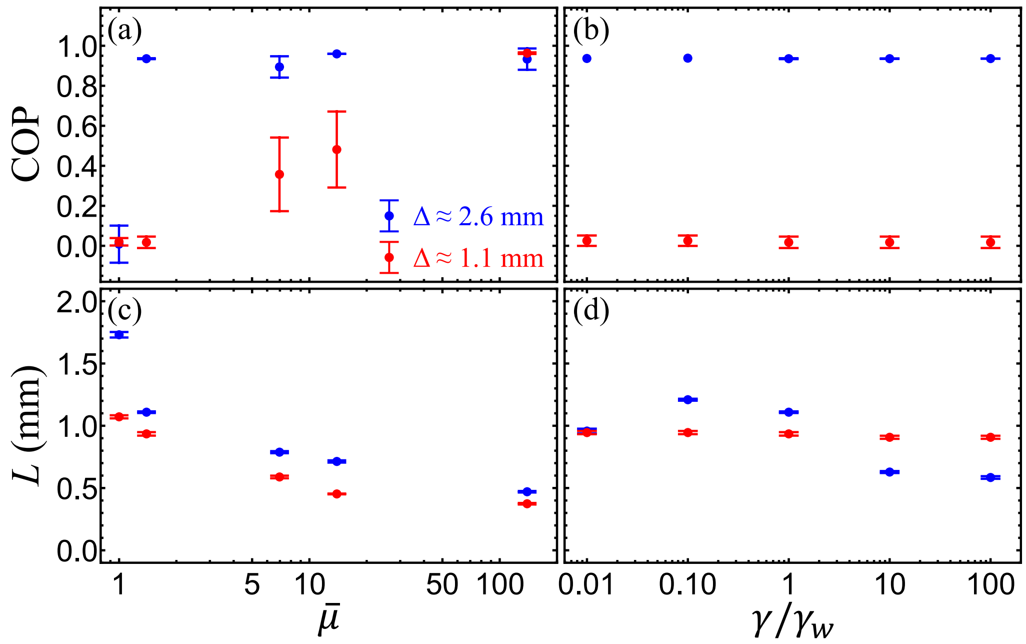

Supplementary Fig. S5: A simulation comparing the role of viscosity contrast and interfacial tension in intradroplet flows and active fluid–oil flow coupling for droplets ($r = 2.4$ mm, $h = 2$ mm) immersed in a thick oil layer (blue dots; $\Delta = 2.6$ mm) or a thin oil layer (red dots; $\Delta = 1.1$ mm). The y-axes are time-averaged circulation order parameter (COP) and cross-correlation length (L) and the x-axes are viscosity contrast, $\bar{\mu} \equiv \mu_o/\mu_w$, and relative interfacial tension, γ/γ_w, where $\gamma_w = 0.072$ N/m is the surface tension of water at 25 °C.[40] Error bars in panels a and b represent standard deviation and error bars in panels c and d represent uncertainty in fitting the correlation function to an exponential function (Fig. 7a inset). The simulation showed that increasing viscosity contrast promoted the formation of intradroplet circulatory flows (panel a) because it suppressed active fluid–oil flow coupling (panel c), whereas interfacial tension played nearly no role in intradroplet flows and flow coupling (panels b&d).

both droplets decayed with viscosity contrast. This decay indicates that increasing the viscosity in oil enhanced energy dissipation, reduced the range of oil that active fluid could drive, and thus suppressed the flow coupling between active fluid and oil. With the suppressed flow coupling, the formation of intradroplet circulation would be dominated by droplet geometry; our previous studies showed that the development of intradroplet circulation requires droplet geometry with an aspect ratio lower than 3, i.e. $r/h \lesssim 3$.[42] The droplets in our simulation had an aspect ratio of $r/h = 1.2$, which met the criteria, so intradroplet circulatory flows were better supported in a system with higher viscosity contrast where flow coupling was suppressed and droplet geometry became the prime factor for circulation development (red dots in Supplementary Fig. S5a).

Interfacial tension. Interfacial tension controls how a droplet will deform in response to external force so as to influence the active fluid–oil coupling and associated intradroplet flows. In our simulation, we neglected this deformation by imposing onto droplets a condition that fixed the droplet geometry, so we hypothesize that in our simulation, the flow coupling and intradroplet active fluid flows will not be affected by a change in interfacial tension. To test this hypothesis within the simulation, we adopted the same pair of droplet systems (Fig. 6a), varied the interface tension γ from 0.00072 to 7.2 N/m or relative interfacial tension γ/γ_w from 0.01 to 100 where $\gamma_w = 0.072$ N/m is surface tension of water at 25 °C, and analyzed the time-averaged COP (Supplementary Fig. S5b). Our analyses showed that the COP did not vary with interfacial tension, which indicates that the intradroplet active fluid flows were not influenced by interfacial tension. To test if the interfacial tension affected the flow coupling between active fluid and oil, we analyzed the cross-correlation length between the active fluid and oil (Supplementary Fig. S5d), revealing that in both droplet systems, the cross-correlation length remained nearly unchanged (within ~20% variation) as we varied the interfacial tension. These analyses demonstrated that the interfacial tension played no role in flow coupling and intradroplet active fluid flows. However, this result was the consequence of assuming a shape-fixed droplet. Experimental results have shown that the droplet deforms more easily as the interfacial tension is lowered,[43] so varying interfacial tension is expected to influence active fluid–oil flow coupling and intradroplet flows. Investigating the role of interfacial tension in active droplet system requires experiments that reduce interfacial tension so that the interfacial fluctuation can be observed, and simulations where the droplet deformation is permitted.[3,44]

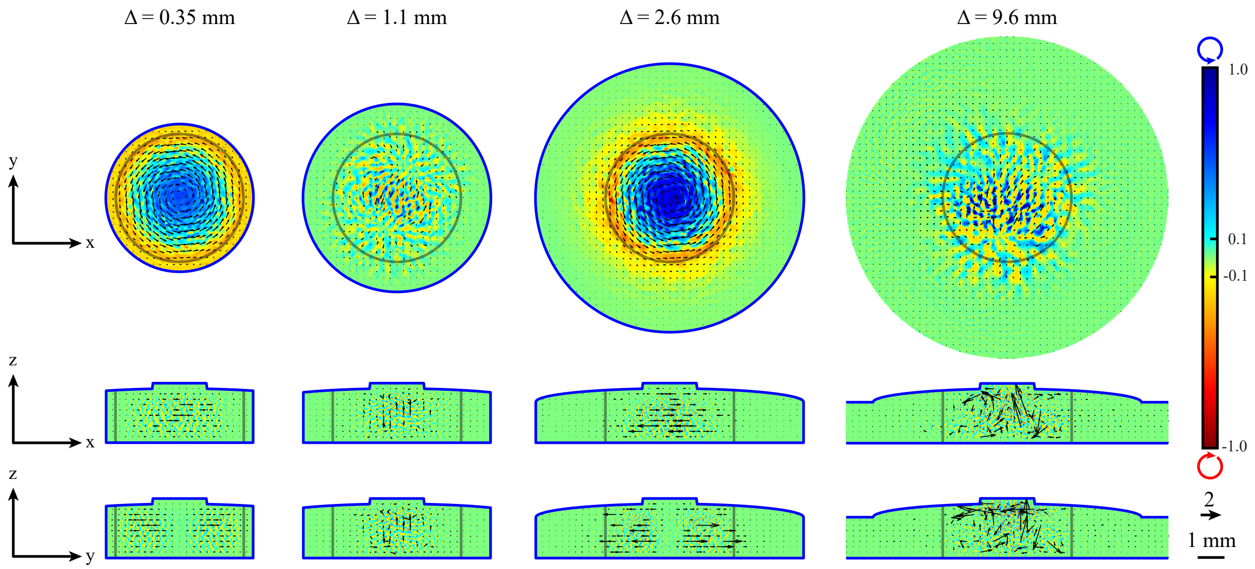

Supplementary Fig. S6: Simulated instantaneous cross-sectional velocity fields and vorticity maps in active droplets that had the same geometry ($r = 2.4$ mm, $h = 2$ mm) but were immersed in oil layers of different thicknesses (Δ =0.35–9.6 mm). The velocity fields and vorticity maps were plotted as in Fig. 1c. The columns from left to right represent various oil layer thicknesses. (The fourth column plots show only the central portion [12 mm wide] of the system.) The rows from top to bottom represent cross-sections at the xy midplane ($z = h/2$), xz midplane ($y = 0$), and yz midplane ($x = 0$), with the axis origin at the bottom center of each droplet. These plots show not only that oil layer thickness influenced intradroplet active fluid flow, but also that when the droplet is in the circulation state, the fluid mainly flows horizontally, whereas when the droplet is in the chaotic state, the fluid flows both horizontally and vertically.

Supplementary Discussion S5: Formation of two-dimensional nematic layers at water–oil interfaces

Microtubule-based active fluid systems are known to deposit microtubules onto the water–oil interface and form 2D active nematics.[45-53] Therefore, we expected microtubules to gather at the water–oil interface and develop 2D active nematics. To verify this expectation, we prepared a compressed water-in-oil active droplet ($r \approx 2.4$ mm, $h = 1$ mm, $\Delta \approx 2.4$ mm) and imaged the microtubules at the water–oil interface of the droplet bottom with fluorescence microscopy (there is a ~1-μm layer of oil between the droplet and the glass plate[48]; Supplementary Fig. S7a). The microtubules were labeled with Alexa 647, which can be imaged with a Cy5 filter cube (excitation: 618–650 nm, emission: 670–698 nm, Semrock, 96376) (Supplementary Fig. S7b&c). The images showed that the microtubules formed a layer of nematics at the water–oil interface with multiple motile plus- and minus-half defects.[16,23,24,48-50,52-55] These motile microtubule-based defects served as a dynamic boundary that confined the active fluid. To examine whether such a dynamic boundary was coupled to the self-organization of the confined active fluid, we simultaneously imaged microtubules at the bottom interface and at the droplet midplane for 15 minutes and then analyzed microtubule motion with the particle image velocimetry algorithm to extract the velocity fields of the microtubule motions (arrows in Supplementary Fig. S7b).[56] The velocity fields enabled us to analyze the circulation order parameter (COP) as a function of time (Supplementary Fig. S7d). Our analyses showed that microtubules at the midplane developed circulatory flows (COP ≈ 0.5, solid blue curve in Supplementary Fig. S7d), whereas at the bottom interface the microtubule flows were chaotic (|COP| $\lesssim$ 0.2, dashed blue curve in Supplementary Fig. S7d). This comparison indicated that the microtubule flows in bulk and at the interface were not coupled. To confirm such bulk–interface decoupling, we repeated the

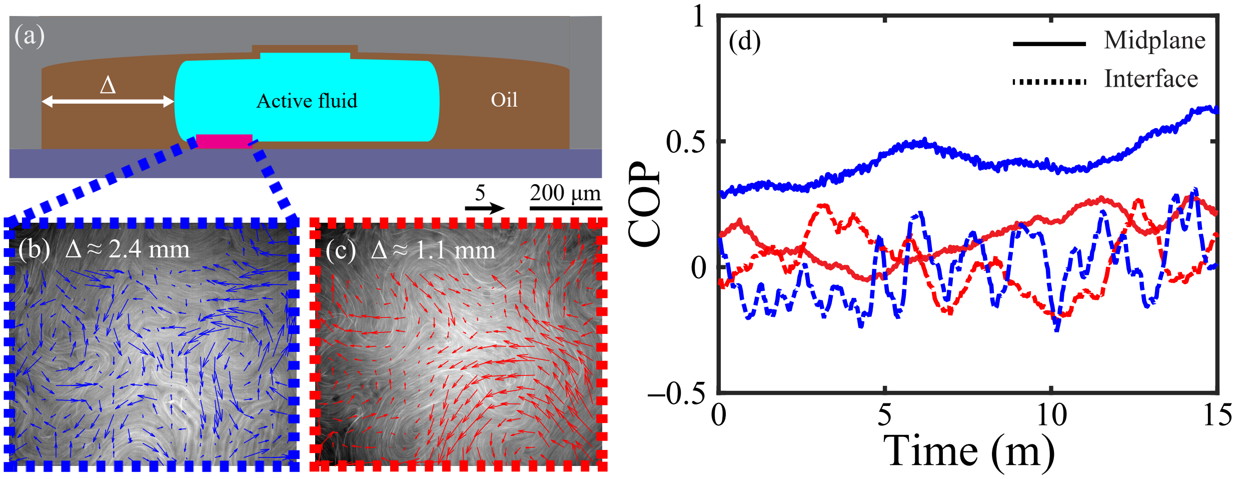

Supplementary Fig. S7: At water–oil interfaces at the bottom of the droplet, microtubules self-organized into a layer of motile nematics whose flows were not influenced by microtubule flows in the droplet bulk. (a) Schematic of imaging the nematic layer at the water–oil interface at the droplet bottom (magenta line). (b&c) Fluorescent micrographs of the microtubule-based nematic layer at the water–oil interfaces of droplets that had the same shape ($r = 2.4$ mm, $h = 1$ mm) but were immersed in oil layers of different thicknesses (Δ). The arrows are normalized velocity fields of corresponding instant nematic flows. (d) Evolution of circulation order parameter (COP) of microtubule flows at the midplane (solid curves) and at water–oil interface (dashed curves). Blue curves represent the droplet with a thick oil layer ($\Delta \approx 2.4$ mm) that developed circulatory flows in bulk (COP ≈ 0.5, solid blue curve) and red curves represent the droplet with a thinner oil layer ($\Delta \approx 1.1$ mm) that developed chaotic flows in bulk (COP≈ 0, solid red curve). However, regardless of how the microtubules flowed in bulk, the microtubule-based nematics flowed chaotically at the interface at the bottom of the droplet (dashed curves).

experiments but immersed the droplet in a thinner oil layer ($\Delta \approx 1.1$ mm, Supplementary Fig. S7c) that did not support circulatory flows in the droplet bulk ($|\mathrm{COP}| \lesssim 0.2$, solid red curve in Supplementary Fig. S7d), and we found that the microtubule motion at the interface remained chaotic ($|\mathrm{COP}| \lesssim 0.2$, dashed red curve in Supplementary Fig. S7d). Our analyses showed that microtubule motion at the interface of the droplet bottom were not related to microtubule motions in bulk. This work demonstrates that microtubules developed 2D active nematics at the water–oil interfaces of droplets and that nematic motions at the droplet bottom interfaces were decoupled from flows in the droplet bulk.

However, our analyses did not suggest that the dynamic boundary was decoupled from the self-organization of intradroplet flows. We have shown that oil near the lateral interface flowed faster when the active fluid flowed chaotically than when the active fluid developed circulatory flows (Fig. 5d), which implies that the microtubule motion at the droplet lateral interface was coupled to the intradroplet fluid flows. Unveiling such a coupling would require further studies monitoring the microtubules at the lateral droplet interfaces.

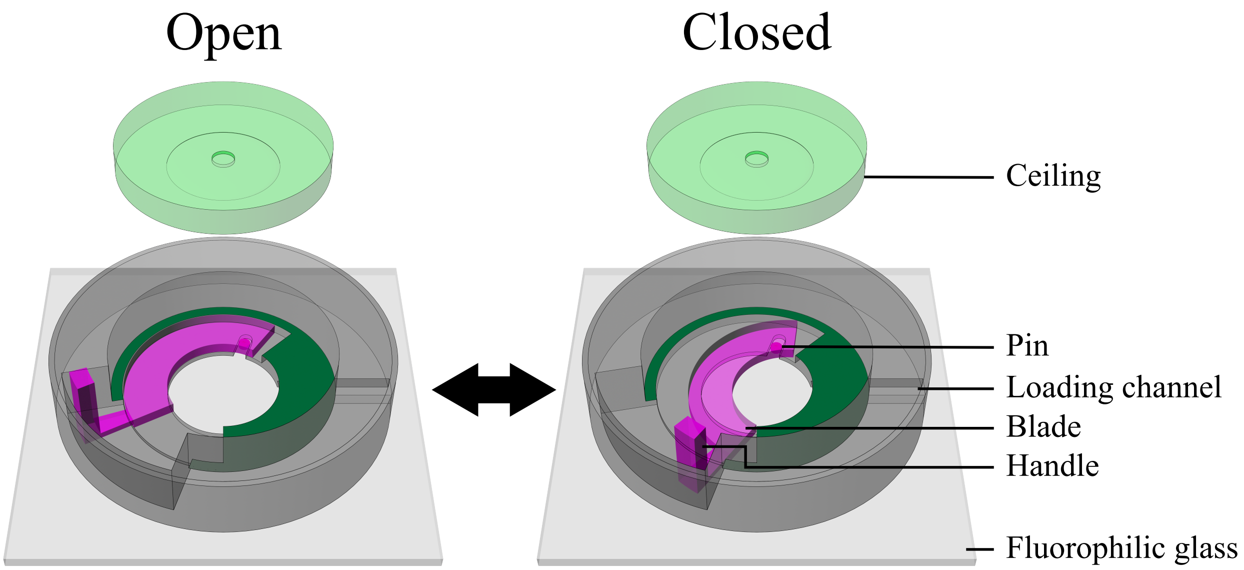

Supplementary Fig. S8: Schematics of the wall-movable milli-fluidic device used to manipulate the layer thicknesses of the oil that immersed an active droplet. The device consisted of a container (gray) that had a cylindrical chamber (radius R = 5 mm, height 1.5 mm), a curved ceiling (R_a = 0.5 mm, $R_b = R$ = 5 mm, light green) that sat on the dark-green platform to enclose the chamber, and a blade (pink) that altered the oil layer thickness. The blade comprised a blade body whose inner side wall served as a movable boundary of the chamber, a pin that was constrained in a groove of the container, and a handle used to manually rotate the blade body around the pin during experiments. Rotating the blade counterclockwise slid the pin inward, shifted the blade midpoint toward chamber center by 1.4 mm, and thus shrank the chamber (left to right). Conversely, rotating the blade clockwise slid the pin outward, shifted the blade midpoint away from the chamber center, and expanded the chamber (right to left). This device enabled real-time tuning of the layer thickness of the oil that immersed the droplets.

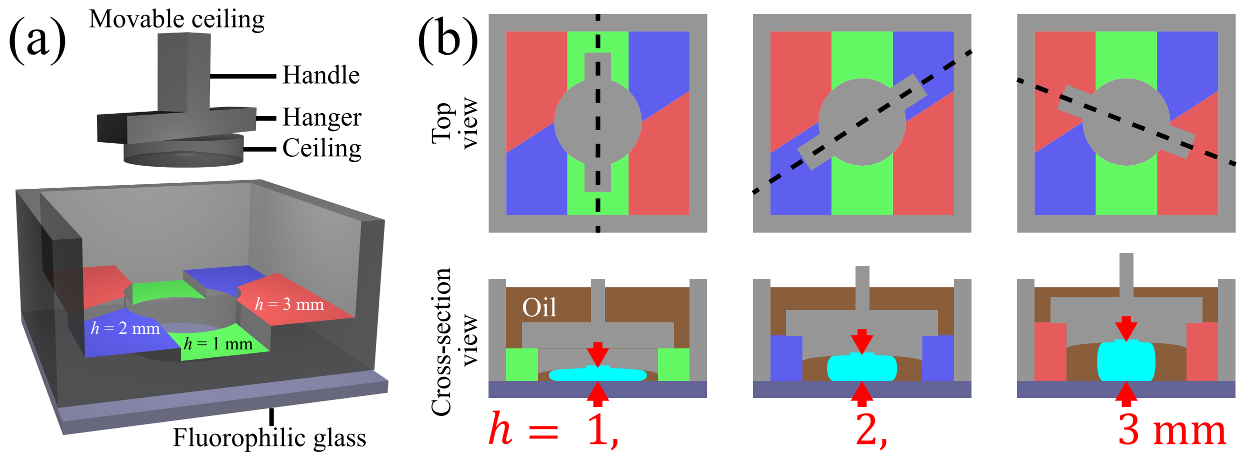

Supplementary Fig. S9: Millifluidic device with movable ceiling for manually compressing a droplet. (a) The device contains a cylindrical well (radius $R = 5$ mm) with a movable ceiling. The ceiling consists of a handle used to manually adjust the ceiling height, a curved ceiling ($R_a = 0.5$ mm, $R_b = R = 5$ mm) used to fix the droplet at the ceiling center, and a hanger that was designed to sit on the colored platforms (green, blue, and red) to place the ceiling at various heights (h). To ensure that the droplet remained in oil while moving the ceiling, we submerged the central well, ceiling, and platforms in oil that was held in a cubical container (gray walls). The front side of the container was transparentized to visualize the platform design. (b) The ceiling hanger was placed on green, blue, and red platforms, causing the ceiling to compress the water-in-oil droplet (cyan) with the heights of h = 1, 2, and 3 mm, respectively. The dashed lines indicate cross-section planes.

Supplementary Video S1: Circulation of microtubule-based active fluids confined in a water-in-oil droplet. The droplet was compressed into a cylinder-like shape with a height of 2 mm and a radius of 1 mm. The time stamp is hour:minute:second.

Supplementary Video S2: The oil layer thickness controlled the formation of circulatory flows in an active fluid droplet, shown by tracer movements. The droplet was compressed into a cylinder-like shape with a height of 2 mm and a radius of 2.4 mm and immersed in an oil bath. When the oil had a layer thickness of 2.6 mm, circulatory flows developed. Decreasing the oil layer thickness to 1.1 mm suppressed the circulatory flows. The time stamp is hour:minute:second.

Supplementary Video S3: A novel millifluidic device manipulated circulatory flows within a water-in-oil active fluid droplet without directly contacting the droplet. The device has one movable wall. Circulatory flows developed after the blade was moved away from the droplet, which increased the oil layer thickness from 1.2 to 2.6 mm (00:33:54–00:34:01). The circulatory flows faded away after the blade approached the droplet, which decreased the oil layer thickness from 2.6 to 1.2 mm (02:00:51–02:00:54). The time stamp is hour:minute:second.

Supplementary Video S4: A novel millifluidic device with a movable ceiling controlled formation of intradroplet circulatory flows. The circulatory flows were suppressed by lifting the ceiling from 2 to 3 mm (00:50:18–00:50:38), whereas the circulatory flows were triggered by lowering the ceiling from 3 to 2 mm (00:30:20–00:30:52). During the times when the ceiling was being moved, the image brightness was oversaturated by the room light that was needed to manually lower or lift the ceiling. The time stamp is hour:minute:second.

Supplementary References

1 Sanchez, T., Chen, D. T. N., DeCamp, S. J., Heymann, M. & Dogic, Z. Spontaneous motion in hierarchically assembled active matter. *Nature* **491**, 431-434 (2012).

2 Tjhung, E., Marenduzzo, D. & Cates, M. E. Spontaneous symmetry breaking in active droplets provides a generic route to motility. *Proceedings of the National Academy of Sciences* **109**, 12381-12386 (2012).

3 Gao, T. & Li, Z. Self-driven droplet powered by active nematics. *Physical Review Letters* **119**, 108002 (2017).

4 Loisy, A., Eggers, J. & Liverpool, T. B. Tractionless self-propulsion of active drops. *Physical Review Letters* **123**, 248006 (2019).

5 Vicsek, T., Czirók, A., Ben-Jacob, E., Cohen, I. & Shochet, O. Novel type of phase transition in a system of self-driven particles. *Physical Review Letters* **75**, 1226-1229 (1995).

6 Chaté, H., Ginelli, F., Grégoire, G., Peruani, F. & Raynaud, F. Modeling collective motion: variations on the Vicsek model. *The European Physical Journal B* **64**, 451-456 (2008).

7 Saintillan, D. & Shelley, M. J. Orientational order and instabilities in suspensions of self-locomoting rods. *Physical Review Letters* **99**, 058102 (2007).

8 Hernandez-Ortiz, J. P., Stoltz, C. G. & Graham, M. D. Transport and collective dynamics in suspensions of confined swimming particles. *Physical Review Letters* **95**, 204501 (2005).

9 Saintillan, D. & Shelley, M. J. Active suspensions and their nonlinear models. *Comptes Rendus Physique* **14**, 497-517 (2013).

10 Saintillan, D. & Shelley, M. J. Instabilities, pattern formation, and mixing in active suspensions. *Physics of Fluids* **20**, 123304 (2008).

11 Saintillan, D. & Shelley, M. J. Instabilities and pattern formation in active particle suspensions: Kinetic theory and continuum simulations. *Physical Review Letters* **100**, 178103 (2008).

12 Gao, T., Blackwell, R., Glaser, M. A., Betterton, M. D. & Shelley, M. J. Multiscale polar theory of microtubule and motor-protein assemblies. *Physical Review Letters* **114**, 048101 (2015).

13 Chen, S., Gao, P. & Gao, T. Dynamics and structure of an apolar active suspension in an annulus. *Journal of Fluid Mechanics* **835**, 393-405 (2018).

14 Wensink, H. H. *et al.* Meso-scale turbulence in living fluids. *Proceedings of the National Academy of Sciences* **109**, 14308-14313 (2012).

15 Dunkel, J. *et al.* Fluid dynamics of bacterial turbulence. *Physical Review Letters* **110**, 228102 (2013).

16 Shankar, S. & Marchetti, M. C. Hydrodynamics of active defects: From order to chaos to defect ordering. *Physical Review X* **9**, 041047 (2019).

17 Bratanov, V., Jenko, F. & Frey, E. New class of turbulence in active fluids. *Proceedings of the National Academy of Sciences* **112**, 15048-15053 (2015).

18 Toner, J., Tu, Y. & Ramaswamy, S. Hydrodynamics and phases of flocks. *Annals of Physics* **318**, 170-244 (2005).

19 Toner, J. & Tu, Y. Flocks, herds, and schools: A quantitative theory of flocking. *Physical Review E* **58**, 4828-4858 (1998).

20 Ramaswamy, S. The mechanics and statistics of active matter. *Annual Review of Condensed Matter Physics* **1**, 323-345 (2010).

21 Aditi Simha, R. & Ramaswamy, S. Hydrodynamic fluctuations and instabilities in ordered suspensions of self-propelled particles. *Physical Review Letters* **89**, 058101 (2002).

22 Thampi, S. P. & Yeomans, J. M. Active turbulence in active nematics. *The European Physical Journal Special Topics* **225**, 651-662 (2016).

23 Thampi, S. P., Golestanian, R. & Yeomans, J. M. Vorticity, defects and correlations in active turbulence. *Philosophical Transactions of the Royal Society A: Mathematical, Physical and Engineering Sciences* **372**, 20130366 (2014).

24 Giomi, L. Geometry and topology of turbulence in active nematics. *Physical Review X* **5**, 031003 (2015).

25 Urzay, J., Doostmohammadi, A. & Yeomans, J. M. Multi-scale statistics of turbulence motorized by active matter. *Journal of Fluid Mechanics* **822**, 762-773 (2017).
26 Cahn, J. W. & Hilliard, J. E. Free energy of a nonuniform system. I. Interfacial free energy. *The Journal of Chemical Physics* **28**, 258-267 (1958).
27 Anderson, D. M., McFadden, G. B. & Wheeler, A. A. Diffuse-interface methods in fluid mechanics. *Annual Review of Fluid Mechanics* **30**, 139-165 (1998).
28 Woodhouse, F. G. & Goldstein, R. E. Spontaneous circulation of confined active suspensions. *Physical Review Letters* **109**, 168105 (2012).
29 Doi, M. & Edwards, S. F. *The Theory of Polymer Dynamics*. (Clarendon Press, 2013).
30 Gao, T., Blackwell, R., Glaser, M. A., Betterton, M. D. & Shelley, M. J. Multiscale modeling and simulation of microtubule--motor-protein assemblies. *Physical Review E* **92**, 062709 (2015).
31 Ezhilan, B., Shelley, M. J. & Saintillan, D. Instabilities and nonlinear dynamics of concentrated active suspensions. *Physics of Fluids* **25**, 070607 (2013).
32 Jeffery, G. B. & Filon, L. N. G. The motion of ellipsoidal particles immersed in a viscous fluid. *Proceedings of the Royal Society of London. Series A, Containing Papers of a Mathematical and Physical Character* **102**, 161-179 (1922).
33 Gao, T., Betterton, M. D., Jhang, A.-S. & Shelley, M. J. Analytical structure, dynamics, and coarse graining of a kinetic model of an active fluid. *Physical Review Fluids* **2**, 093302 (2017).
34 Chaikin, P. M. & Lubensky, T. C. *Principles of Condensed Matter Physics*. Vol. 1 (Cambridge University Press, 2000).
35 Mittal, S. & Kumar, B. A stabilized finite element method for global analysis of convective instabilities in nonparallel flows. *Physics of Fluids* **19**, 088105 (2007).
36 TaoHe. An efficient selective cell-based smoothed finite element approach to fluid-structure interaction. *Physics of Fluids* **32**, 067102 (2020).
37 Holzbecher, E. Convection pattern formation in a domain with a horizontal interface. *Physics of Fluids* **31**, 056602 (2019).
38 Bendaraa, A., Charafi, M. M. & Hasnaoui, A. Numerical study of natural convection in a differentially heated square cavity filled with nanofluid in the presence of fins attached to walls in different locations. *Physics of Fluids* **31**, 052003 (2019).
39 Li, Q., Ito, K., Wu, Z., Lowry, C. S. & Loheide II, S. P. COMSOL Multiphysics: A novel approach to ground water modeling. *Groundwater* **47**, 480-487 (2009).
40 Gittens, G. J. Variation of surface tension of water with temperature. *Journal of Colloid and Interface Science* **30**, 406-412 (1969).
41 Rausch, M. H., Kretschmer, L., Will, S., Leipertz, A. & Fröba, A. P. Density, surface tension, and kinematic viscosity of hydrofluoroethers HFE-7000, HFE-7100, HFE-7200, HFE-7300, and HFE-7500. *Journal of Chemical & Engineering Data* **60**, 3759-3765 (2015).
42 Wu, K.-T. *et al.* Transition from turbulent to coherent flows in confined three-dimensional active fluids. *Science* **355**, eaal1979 (2017).
43 Keber, F. C. *et al.* Topology and Dynamics of Active Nematic Vesicles. *Science* **345**, 1135-1139 (2014).
44 Young, Y.-N., Shelley, M. J. & Stein, D. B. The many behaviors of deformable active droplets. *Mathematical Biosciences and Engineering* **18**, 2849-2881 (2021).
45 Sanchez, T. *et al.* Combined noninvasive metabolic and spindle imaging as potential tools for embryo and oocyte assessment. *Human Reproduction* **34**, 2349-2361 (2019).
46 Lemma, L. M. *et al.* Multiscale Dynamics in Active Nematics. arXiv:2006.15184 [cond-mat.soft] (2020).
47 Lemma, L. M., Decamp, S. J., You, Z., Giomi, L. & Dogic, Z. Statistical properties of autonomous flows in 2D active nematics. *Soft Matter* **15**, 3264 (2019).
48 DeCamp, S. J., Redner, G. S., Baskaran, A., Hagan, M. F. & Dogic, Z. Orientational order of motile defects in active nematics. *Nature Materials* **14**, 1110-1115 (2015).

49 Opathalage, A. *et al.* Self-organized dynamics and the transition to turbulence of confined active nematics. *Proceedings of the National Academy of Sciences* **116**, 4788-4797 (2019).

50 Norton, M. M. *et al.* Insensitivity of active nematic liquid crystal dynamics to topological constraints. *Physical Review E* **97**, 012702 (2018).

51 Guillamat, P., Ignés-Mullol, J., Shankar, S., Marchetti, M. C. & Sagués, F. Probing the shear viscosity of an active nematic film. *Physical Review E* **94**, 060602 (2016).

52 Guillamat, P., Ignés-Mullol, J. & Sagués, F. Taming active turbulence with patterned soft interfaces. *Nature Communications* **8**, 564 (2017).

53 Guillamat, P., Hardoüin, J., Prat, B. M., Ignés-Mullol, J. & Sagués, F. Control of active turbulence through addressable soft interfaces. *Journal of Physics: Condensed Matter* **29**, 504003 (2017).

54 Putzig, E., Redner, G. S., Baskaran, A. & Baskaran, A. Instabilities, defects, and defect ordering in an overdamped active nematic. *Soft Matter* **12**, 3854-3859 (2016).

55 Doostmohammadi, A., Ignés-Mullol, J., Yeomans, J. M. & Sagués, F. Active nematics. *Nature Communications* **9**, 3246 (2018).

56 Thielicke, W. & Stamhuis, E. J. PIVlab–Towards user-friendly, affordable and accurate digital particle image velocimetry in MATLAB. *Journal of Open Research Software* **2**, e30 (2014).
